# The ACE2 Receptor for Coronavirus Entry Is Localized at Apical Cell—Cell Junctions of Epithelial Cells

**DOI:** 10.3390/cells11040627

**Published:** 2022-02-11

**Authors:** Florian Rouaud, Isabelle Méan, Sandra Citi

**Affiliations:** Department of Cell Biology, Faculty of Sciences, University of Geneva, 1205 Geneva, Switzerland; florian.rouaud@unige.ch (F.R.); isabelle.mean@unige.ch (I.M.)

**Keywords:** ACE2, coronavirus, Cell—Cell junctions, epithelium

## Abstract

Transmembrane proteins of adherens and tight junctions are known targets for viruses and bacterial toxins. The coronavirus receptor ACE2 has been localized at the apical surface of epithelial cells, but it is not clear whether ACE2 is localized at apical Cell—Cell junctions and whether it associates with junctional proteins. Here we explored the expression and localization of ACE2 and its association with transmembrane and tight junction proteins in epithelial tissues and cultured cells by data mining, immunoblotting, immunofluorescence microscopy, and co-immunoprecipitation experiments. ACE2 mRNA is abundant in epithelial tissues, where its expression correlates with the expression of the tight junction proteins cingulin and occludin. In cultured epithelial cells ACE2 mRNA is upregulated upon differentiation and ACE2 protein is widely expressed and co-immunoprecipitates with the transmembrane proteins ADAM17 and CD9. We show by immunofluorescence microscopy that ACE2 colocalizes with ADAM17 and CD9 and the tight junction protein cingulin at apical junctions of intestinal (Caco-2), mammary (Eph4) and kidney (mCCD) epithelial cells. These observations identify ACE2, ADAM17 and CD9 as new epithelial junctional transmembrane proteins and suggest that the cytokine-enhanced endocytic internalization of junction-associated protein complexes comprising ACE2 may promote coronavirus entry.

## 1. Introduction

The apical junctional complex of epithelial cells consists of tight junctions (TJ) and adherens junctions (AJ), and TJ forms a critical barrier to the entry of pathogens into the organism [1]. Several viruses, including adenovirus, coxsackievirus, reovirus, hepatitis C virus, measles virus, herpes virus and polioviruses exploit transmembrane TJ and AJ proteins, such as JAM-A, CAR, nectins and claudins to gain access to the cell interior and spread in tissues (reviewed in [2,3,4,5]). Furthermore, several bacterial toxins, including *Clostridium perfringens* enterotoxin and *Staphylococcus aureus* α-toxin bind to transmembrane junctional proteins of TJ and AJ, such as claudins [6] and ADAM10 [7], and affect epithelial junction integrity to exert their toxic effects (reviewed in [5,8,9]). However, it is not clear whether coronaviruses use transmembrane junctional proteins as receptors and gateways for entry into cells.

The Angiotensin-Converting-Enzyme-2 (ACE2) is the main receptor for coronavirus entry [10,11,12,13,14,15] and is a single-pass transmembrane protein with an extracellular N-terminal domain and an intracellular C-terminal tail [16]. Transcript, immunocytochemistry and immunofluorescence microscopy (IF) analyses show that ACE2 is expressed in epithelial cells of human airways, olfactory, kidney, intestinal tissues, and in endothelial cells [17,18,19,20,21,22]. ACE2 interacts with several proteins, which may control its localization and function. For example, ACE2 interacts with ADAM17 (A Disintegrin And Metalloproteinase 17), which regulates the proteolytic shedding of the ACE2 ectodomain [23,24,25,26,27,28,29] and is also required for the infection of epithelial cells by oncogenic papillomaviruses, through the formation of a surface platform comprising the tetraspanin CD151 and the epidermal growth factor receptor (EGFR) [30]. Proteolytic priming of Coronaviruses and influenza virus occurs in tetraspanin-enriched membrane microdomains, and the tetraspanin CD9 facilitates binding of MERS (Middle East Respiratory Syndrome) coronavirus to the cell surface [31,32] and associates with ADAM17 on the surface of leukocytes and endothelial cells [33]. Together, these observations suggest that multiprotein complexes comprising ACE2, ADAM17 and CD9 could mediate entry of coronaviruses, including SARS-CoV-2, across the plasma membrane of epithelial and other cell types.

Little is known about the subcellular localization, dynamics and mechanisms of localization and internalization of ACE2. Immunofluorescence analysis indicates that ACE2 is localized on the apical surface of differentiated epithelial cells [34,35,36]. Interactions mediated by the C-terminal domain are critical for ACE and ACE2 localization [37]. Importantly, recent evidence shows that PDZ-containing proteins, including the scaffolding protein ZO-1 and the apical polarity complex protein Par3, are targeted by MERS-CoV and SARS-CoV-2 [38,39]. ZO-1 and Par3 are cytoplasmic components of apical junctions, and ZO-1 is one of the main scaffolding proteins that anchor transmembrane proteins at TJ, by connecting their cytoplasmic C-terminal regions to the actin cytoskeleton [40,41,42]. In transfected HEK293 cells, exogenous ACE2 localizes at regions of Cell—Cell contact [43,44]. However, localization of endogenous ACE2 at junctions of epithelial cells has not been reported, and nothing is known about a junctional localization of the ACE2 interactors ADAM17 and CD9 in epithelial cells.

Here, we explored the expression and localization of ACE2, ADAM17 and CD9 in tissues and in cultured epithelial cells and found that they are localized at junctions and co-immunoprecipitate from lysates of cultured epithelial cells, and ACE2 expression correlates with the expression of TJ proteins.

## 2. Materials and Methods

### 2.1. Data Mining

Levels of ACE2, CD9 and ADAM17 mRNA expression (TPM, Transcript Per Million) were obtained by query of the GTEx Portal (https://gtexportal.org/home/multiGeneQueryPage, accessed on 28 January 2022), which collects data from microarray analyses of tissues. Publicly available gene expression data sets from the GEO database (NCBI’s Gene Expression Omnibus) were used to analyze ACE2, ADAM17, CD9, OCLN and CGN (ID_REF: 219962_at, 213532_at, 201005_at, 209925_at and 223233_s_at, respectively) mRNA levels in different cell lines (Gene Expression ID: GSE41445) and Caco-2 cells (Gene Expression ID: GSE7259). For datasets GSE41445 and GSE7259 [45] signal intensities (Y axis) were obtained using Affymetrix GeneChip Operating software (GCOS, version 1.4.0.036; Instrument control version: 6.0.1.002). To examine correlations between mRNA expression levels, values of the indicated genes/mRNAs from the GSE41445 dataset were plotted and analyzed by Spearman’s correlation using Prism v.8.0, to obtain the correlation coefficient (r) and the statistical significance (two-tailed, *p*-value).

### 2.2. Cell Culture

mCCD, A427, A549, and HaCaT were cultured as previously described [46]. Calu-I (human lung carcinoma cell line, a kind gift of Marco Paggi, Regina Elena National Cancer Institute, Italy) [47] were cultured in RPMI (Gibco, PAN^TM^ Biotech Aidenbach Germany) supplemented with 10% inactivated foetal bovine serum (FBS, PAN^TM^ Biotech Aidenbach Germany), 1X minimal essential medium non-essential amino acids (MEM NEAA, PAN Biotech). Eph4 and Hap1 cells were cultured as previously described [7,48,49]. H1299 (human non-small cell lung carcinoma cell line derived from lymph node), MCF7 (human breast adenocarcinoma), MDA-MB-231 (human breast adenocarcinoma), U-2 OS (human osteosarcoma) (a kind gift from Josephine Zangari, University of Geneva, Switzerland), SKCO-15 [50] and HeLa [51] cells were cultured in DMEM (Gibco) supplemented with 10% inactivated foetal bovine serum (FBS, PAN Biotech).

### 2.3. Antibodies

The following primary antibodies against the indicated proteins were used for immunoblotting (IB) and immunofluorescence microscopy (IF) at the indicated dilutions: monoclonal mouse anti-ZO-1 (Thermo Fischer Scientific, Waltham, MA USA, 33–9100, 1:2000 IB), monoclonal mouse anti-cingulin (in house 22BD5A1, 1:5000 IB and 1:1000 IF) [46], polyclonal rabbit anti-ACE2 (Abcam, ab15348, 1:500 IB and 1:100 IF), polyclonal rabbit anti-CD9 (Abcam, ab92726, 1:500 IB and 1:100 IF) and polyclonal goat anti-ADAM17 (Abcam, ab13535, 1:500 IB and 1:100 IF). Anti-mouse and anti-rabbit (1:20000, Promega AG, Dübendorf Switzerland, W402B and W401B, respectively), and anti-goat (1:10000, Thermo Fisher Scientific, 62-9520) IgG HRP-conjugated antibodies were used for IB. Secondary antibodies for IF were anti-mouse Cy5 (against mouse anti-CGN), anti-rabbit Alexa Fluor 488 (against anti-ACE2/CD9), anti-goat Cy3 (against anti-ADAM17) (Jackson ImmunoResearch Europe, Newmarket, UK) (1:300).

### 2.4. Preparation of Lysates, SDS-PAGE and Immunoblotting

For the preparation of cell lysates, cells were grown for 72 h on 10 cm plastic dishes, washed with cold PBS, lysed with RIPA buffer (NaCl 1.5 M, Tris-HCl pH 7.5 50 mM, Triton 0.5%, Glycerol 10%, EDTA 10 mM, 0.1% SDS, 2.5% Deoxycholic Acid) and Roche Protease Inhibitor Cocktail (1×) at 4 °C, and incubated for 15 min at 4 °C with gentle agitation. Lysates were sonicated 5 s at 66% power (3 bursts, 4 °C), centrifuged for 20 min at 13,000 rpm, and supernatants were recovered [46]. Samples (15 μg total protein) were mixed with SDS sample buffer, incubated at 95 °C for 5 min, and subjected to SDS–PAGE (8–12% acrylamide). For immunoblotting (IB), gels were transferred onto nitrocellulose (0.45 μm) (100 V for 80 min at 4 °C), and blots were incubated with primary antibody (16 h at 4 °C), followed by washing, incubation with secondary HRP-labeled antibody (1 h at RT), washing, and development of ECL luminescence reaction (Amersham^TM^ ECL, Advansta San Jose, CA, USA). For quantifications of IB signals, exposed films were scanned and saved as tiff files, and band profiles were measured using ImageJ and expressed as relative intensity, from the ratio between the signal of the protein of interest and the internal control.

### 2.5. Immunoprecipitation

Immunoprecipitation was carried out as described previously [52,53]. The supernatants of the cytoskeleton-soluble and -insoluble fractions were combined to obtain the total cell lysate. 20 µL of Dynabeads protein G (Invitrogen 100.04D) were coupled to antibodies (diluted in PBS/BSA 5%; 1/100 of anti-GFP, anti-VE-Cadherin, anti-Ace2 anti-ADAM17 and anti-CD9) at 4 °C for 90 min. After two washes with PBS/BSA 5%/Nonidet P-40 1%, beads were incubated overnight at 4 °C with 100 µL of total cell lysate and then washed three times with co-immunoprecipitation (CoIP) buffer (150 mM NaCl, 20 mM Tris-HCl, pH 7.5, 1% Nonidet P-40, 1 mM EDTA, 5 mg/mL antipain, 5 mg/mL leupeptin, 5 mg/mL pepstatin, 1 mM PMSF). Immunoprecipitates were eluted in 20 µL SDS loading buffer and boiled 5 min at 95 °C, before analysis by SDS-PAGE and immunoblotting.

### 2.6. Immunofluorescence

Confluent monolayers were fixed with cold methanol for 10 min at −20 °C, incubated with primary antibodies overnight at 4 °C, washed 3 times for 10 min with PBS, incubated with fluorophore conjugated secondary antibodies for 30 min at 37 °C, and washed 3 times for 10 min with PBS. Coverslips were mounted with Vectashield containing DAPI (Reactolab, S.A. Servion Switzerland) and observed with a Zeiss LSM800 confocal microscope using a Plan-Apochromat 63×/1.40 oil objective at a resolution of 1024 × 1024 px with maximum intensity projections of z-stack images (typically 4 confocal planes over 1.2 µm, step size = 0.3). Images were extracted from lif., lsm. or czi. files using ImageJ, adjusted and cropped using Adobe Photoshop, and assembled in Adobe Illustrator figures.

## 3. Results

### 3.1. ACE2 mRNA Expression in Epithelial Tissues and Cells Correlates with CD9 and Tight Junction Proteins and Is Up-Regulated upon Epithelial Differentiation

**Figure 1:**
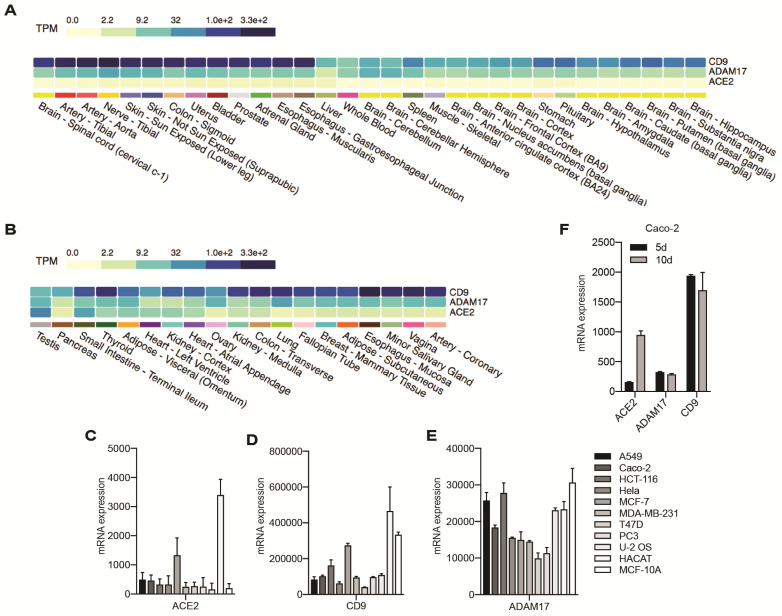
Analysis of mRNA expression levels for ACE2, ADAM17 and CD9 in tissues and cells. (**A**,**B**) Analysis of datasets from GTexPortal (https://www.gtexportal.org/home/multiGeneQueryPage/ACE2,ADAM17,CD9, accessed on 28 January 2022). (**A**) shows tissues with relatively lower expression of ACE2 and (**B**) shows tissues with relatively medium or high expression of ACE2. TPM refers to Transcript Per Million. Color code (light green to dark blue) correlates with transcript levels (from light green = lowest to dark blue = highest). (**C**–**E**) Signal intensities (absolute mRNA expression) of ACE2 (**C**), CD9 (**D**) and ADAM17 (**E**) in the indicated cultured cell lines (data from GSE41445 microarray datasets). (**F**) Signal intensities for mRNA expression of ACE2, CD9 and ADAM17 (GSE7259 microarray datasets) in human intestinal (colon) carcinoma Caco-2 either of low (5-day culture, black bars) or high (10-day culture, grey bars) differentiation. Note that ACE2 mRNA expression is upregulated upon differentiation.

We first analyzed the expression of ACE2 and its interactors ADAM17 and CD9 at the mRNA level in human tissues, using GTEx Portal datasets. ACE2 expression was lowest in brain, muscle, blood, liver, spleen and other tissues (Figure 1A), and highest, among others, in heart, testis, small intestine and other epithelial tissues (Figure 1B). The strongest co-expression of ACE2, CD9 and ADAM17 mRNAs was found to occur in intestinal, kidney, breast and lung epithelial tissues (Figure 1B). We analyzed whether the tissue mRNA expression levels of ACE2, CD9 and ADAM17 are correlated, and found a correlation of expression between ADAM17 and CD9 (Figure S1A), but not between either ACE2 and ADAM17 or ACE2 and CD9 (Figure S1B,C).

Next, we analyzed the mRNA expression of ACE2, ADAM17 and CD9 in datasets of cultured cell lines, including HaCaT cells (keratinocytes), epithelial carcinoma cell lines, such as Caco-2 (colon), MCF-7, MCF-10A, T47D, MDA-MB-231 (breast), HeLa (uterine), PC3 (prostate), and the non-epithelial cancer cell line U-2 OS (osteosarcoma). ACE2 mRNA expression was highest in HaCaT and MCF-7 cells, which also showed high CD9 and ADAM17 expression (Figure 1C–E). Since epithelial tissues and cells are characterized by the presence of tight junctions (TJ), we also evaluated the expression of the TJ proteins cingulin (CGN) and occludin (OCLN). In tissues, the strongest co-expression of ACE2 mRNA with occludin and cingulin was observed in epithelial tissues, including the intestine, kidney, oesophagus, thyroid and lung (Figure S1D). In cultured cells, occludin and cingulin expression was highest in Caco-2 cells and lowest in HeLa cells (Figure S1E) (see also [51]). Importantly, ACE2 levels of mRNA expression in cultured cell lines correlated with occludin and cingulin (Figure S1F,G). Moreover, in Caco-2 cells, ACE2 expression levels increased in correlation with Caco-2 differentiation, which is also associated with an increase in the mRNA levels of TJ proteins [54], whereas expression of ADAM17 and CD9 remained constant (Figure 1F). Together, these results suggest that ACE2 expression correlates with epithelial polarization and increased expression of TJ proteins.

### 3.2. ACE2 Protein Is Expressed in Epithelial Cultured Cells and Co-Immunoprecipitates with ADAM17 and CD9 from Lysates of HaCaT and HeLa Cells

**Figure 2:**
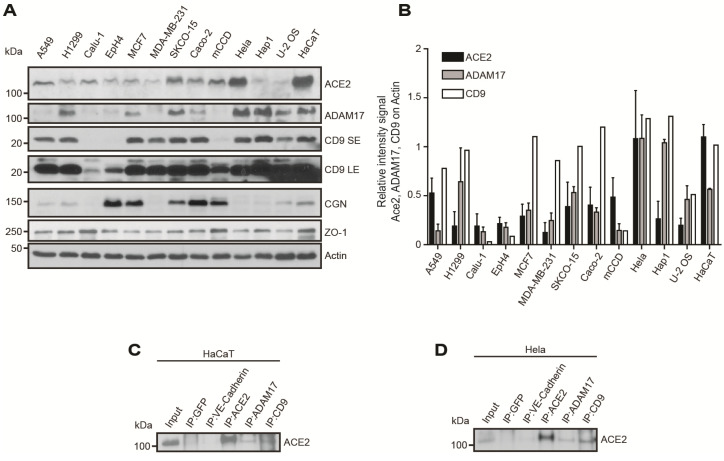
The ACE2 receptor is widely expressed in cultured cell lines and co-immunoprecipitates with ADAM17 and CD9 in lysates of HaCaT and HeLa epithelial cells. (**A**,**B**) Immunoblot (IB) analysis (**A**) and quantification (**B**) (*n* = 2 except for CD9, *n* = 1) of the expression of ACE2, ADAM17 and CD9 in lung epithelial/lung cancer cells (A549, H1299, Calu-I), mammary epithelial (Eph4) and breast adenocarcinoma cells (MCF7, MDA-MB-231), colon adenocarcinoma cells (SKCO-15, Caco-2), kidney collecting duct cells (mCCD), uterine cervical cancer cells (HeLa), myeloblastic haploid cells (Hap1), osteosarcoma cells (U-2 OS), and immortalized keratinocytes (HaCat). IBs for CGN (cingulin) and ZO-1, which are cytoplasmic proteins of tight junction (TJ) (CGN and ZO-1) and adherens junctions (ZO-1), are also shown. IB of actin is shown for the normalization of protein levels. Numbers on the left indicate approximate molecular sizes, based on the migration of prestained markers. SE and LE refer to Short Exposure and Long Exposure, respectively, for CD9. (**C**,**D**) Immunoblot analysis of endogenous ACE2 SARS-CoV-2 receptor (ACE2) in immunoprecipitates (IPs) obtained from lysates of HaCat (**B**) and HeLa (**C**) cells, using the indicated antibodies. Note that ACE2 is detected in IPs of ADAM17 and CD9. Ips obtained using antibodies against GFP and VE-cadherin are negative controls, and IP:ACE2 is a positive control.

Next, we examined the expression of ACE2, ADAM17 and CD9 proteins in epithelial and non-epithelial cultured cell lines by immunoblot (IB) analysis, using as internal reference standards the TJ marker cingulin (CGN), and the TJ/AJ marker ZO-1 (TJP1) (Figure 2A, quantification in Figure 2B). ACE2 was detected in lysates from cell lines originally derived from lung (Calu-1) and lung cancer (A549, H1299), mammary gland (Eph4) and mammary breast cancer (MCF-7, MDA-MB-231), colon cancer (SKCO15, Caco2), kidney collecting duct (mCCD), cervical cancer (HeLa), in myeloblastic-derived haploid fibroblasts (Hap1), in human osteosarcoma (U-2 OS) and epidermal (HaCaT) cells, with highest levels in HeLa and HaCat cells (Figure 2A,B). ADAM17 signal was highest in HeLa and Hap1 cell lysates, and low in A549, Calu-1, Eph4, MDA-MB-231, and mCCD cell lysates (Figure 2A,B). CD9 was detected at similar levels in all cell lines, except for Calu-1, Eph4, and mCCD, where the signal was low and required longer exposure for detection (Figure 2A,B, LE=long exposure). CGN expression was highest in cell lines showing characteristics of well-polarized epithelial cells with TJ, such as Eph4, Caco2 and mCCD, and showed low or undetectable expression in several cancer lines, such as MDA-MB-231, Hela, and U-2OS (Figure 2A,B). ZO-1, on the other hand, was detected in all cell lysates, consistent with its distribution both at TJ in polarized epithelial cells and at AJ of non-polarized/non-epithelial cells (Figure 2A,B). In summary, most cultured epithelial cell lines showed co-expression of ACE2, ADAM17 and CD9 together with CGN and ZO-1.

Next, we asked whether ACE2 is present in a complex with ADAM17 and CD9 in epithelial cells, by carrying out immunoprecipitation (IP) analysis. We selected HeLa and HaCaT cells, that showed the highest ACE2 protein expression. ACE2 was detected in immunoprecipitates (IPs) of endogenous ADAM17 and CD9 both in HaCat (Figure 2C) and HeLa cells (Figure 2D), as well as in ACE2 immunoprecipitates (positive control) but not in IPs of GFP and VE-cadherin (negative controls) (Figure 2C,D). Previously, ADAM17 and CD9 were shown to co-immunoprecipitate and interact together [55]. In summary ACE2, ADAM17 and CD9 are co-expressed and co-immunoprecipitated in epithelial cell lysates, suggesting that they can form a complex.

### 3.3. ACE2, ADAM17 and CD9 Are Localized at Cell—Cell Junctions

**Figure 3:**
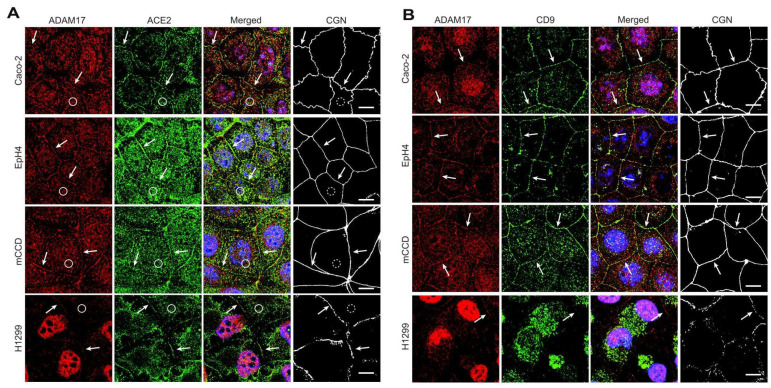
ACE2, ADAM17 and CD9 are localized at apical junctions of cultured epithelial cells. (**A**,**B**) Immunofluorescent microscopy localization of ADAM17, ACE2 and cingulin (CGN) (**A**), ADAM17, CD9 and CGN (**B**) in the indicated cell types (Caco2, Eph4, mCCD and H1299 cells). Arrows indicate junctional localization, circles indicate apical surface labeling, and dashed circles indicate a lack of apical cingulin labeling. CGN is used as an apical junctional reference marker. Bar = 10 microns.

To examine the localization of ACE2 and its transmembrane protein partners in polarized epithelial cells, mCCD, Eph4 and Caco-2 cells were grown on Transwell filters to obtain maximum polarization. Cells were triple-labelled with antibodies, either against ACE2, ADAM17, and the TJ marker cingulin for reference or against ADAM17, CD9 and cingulin. Confocal immunofluorescence microscopy analysis revealed that ACE2 co-localizes with ADAM17 and cingulin at apical junctions of Caco-2 (intestinal), Eph4 (mammary), mCCD (renal) and H1299 (lung) cells (arrows, Figure 3A). Furthermore, ADAM17 co-localized with CD9 and cingulin at apical junctions of the same cells (arrows, Figure 3B). In addition, consistent with previous studies [18,19,22,34,35], we also detected a diffuse apical surface labeling of ACE2 and ADAM17 (circles, Figure 3A), but not cingulin (dashed circles, Figure 3A). Together, these observations demonstrate that a pool of ACE2 is colocalized with CD9 and ADAM17 at apical epithelial junctions. 

## 4. Discussion 

**Figure 4:**
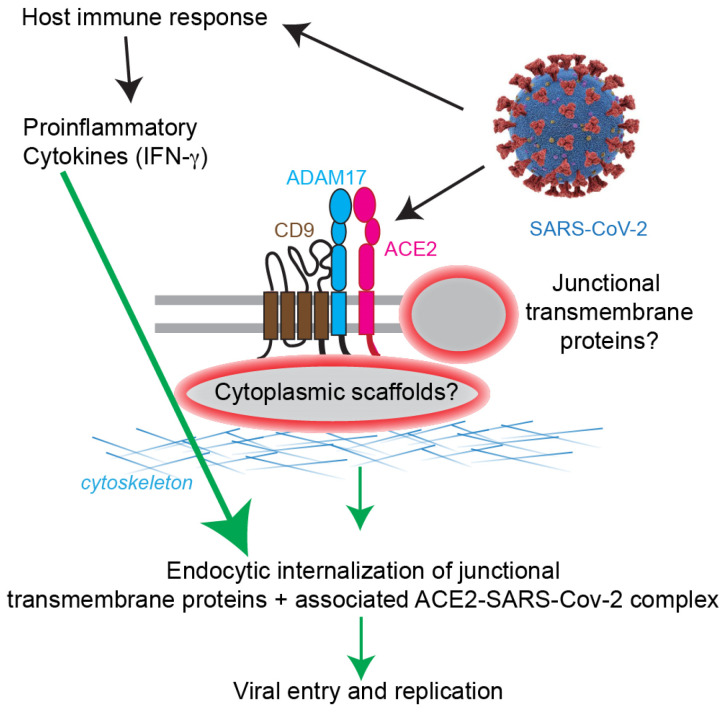
Scheme of working hypothesis. SARS-CoV-2 binds to the host cell receptor ACE2, which forms a complex with ADAM17 and CD9 at epithelial apical junctions. The identity of junctional transmembrane and cytoplasmic proteins associated with the complex and their role in the localization and internalization of ACE2, CD9 and ADAM17 is not known (grey/red ovals and question marks). SARS-CoV-2 elicits a host immune response, which entails the release of pro-inflammatory cytokines, which promotes endocytic internalization of junctional transmembrane proteins and associated complexes (green arrows).

The localization of transmembrane proteins is essential for their function, and here we show that the main coronavirus receptor ACE2, in addition to the previously reported apical localization, is concentrated at epithelial apical Cell—Cell junctions in cultured epithelial cell lines. The interaction of SARS-CoV-2 with ACE2 has important therapeutic implications [56,57], and the junctional localization of ACE2 reported here is relevant to the mechanisms of SARS-CoV-2 internalization and penetration across epithelial barriers. Several pathogens are internalized into cells as part of complexes with surface adhesion and junctional proteins: coronaviruses, like influenza viruses, rhinoviruses, reoviruses, and dengue virus, bypass the epithelial barrier by hijacking receptors and undergoing endocytosis [4,58]. More precisely, SARS coronaviruses are internalized by macropinocytosis [44,59,60]. Moreover, the barrier function of epithelia and endothelia is disrupted by inflammatory mediators, such as interleukins, tumor necrosis factor, and interferon-gamma (IFN-γ) that are released from activated immune cells [4,61,62,63,64]. TNF-α and IFN-γ disrupt epithelial barriers through different mechanisms: cytoskeletal rearrangements [65], disruption of TJ organization [66], internalization of TJ membrane proteins by macropinocytosis [67], RhoA/ROCK-mediated, myosin II-dependent formation of a vacuolar apical compartment [68], activation of MAPK signaling [69], and downregulation of expression of TJ proteins [70]. Interestingly, IFN-γ also induces up-regulation of ACE2 expression [20], suggesting that inflammation could drive not only increased attachment of coronavirus (for example SARS-CoV-2) to cells but also increased internalization of ACE2 and SARS-CoV-2 through micropinocytosis (Figure 4). The critical importance of the apical junctional complex in host-pathogen interactions is also highlighted by the observation that although the *S. aureus* α-toxin receptor ADAM10 is distributed throughout the lateral surfaces of epithelial cells, the AJ-associated pool of ADAM10 is required for maximal toxicity, through clustering and stabilization of the toxin pores [7,49]. The junctional localization of ACE2 in epithelial cells remains to be confirmed in human tissues and mouse models, and the subcellular localization of ACE2 in non-epithelial cell types should also be investigated in further detail. Our study also shows that data mining using GTEx and GEO profile is a helpful and user-friendly tool to investigate gene expression and correlations of expression between genes, and it does not require specialized expertise in microarray analysis or time-consuming download of big datasets.

Little is known about the cytoplasmic scaffolding proteins that anchor ACE2, CD9 and ADAM17 at specific plasma membrane domains. However, this is an important question since intracellular anchoring proteins and membrane microdomains are critically involved in regulating the dynamics and efficiency of pathogen internalization [71,72,73,74,75]. For example, plasma membrane cholesterol- and sphingolipid-rich lipid raft microdomains are organized by tetraspanins and are very important for virus entry [44,76,77,78]. A complex of scaffolding host receptors and priming proteases, such as TMPSSR2 promote viral-cell membrane fusion of coronaviruses [12,31,32,79,80]. Furthermore, scaffolding proteins are targets of viral pathogens [8]. Importantly, the SARS coronavirus protein E interacts with the TJ protein PALS1 to alter TJ formation [81], and recent high throughput affinity profiling experiments show that the C-terminal regions of SARS-CoV-2 proteins E, 3a and N are potential interactors of additional PDZ-containing proteins, such as the TJ/AJ associated proteins ZO-1 and Par3 [38]. Thus, a disruption of the scaffolding complexes that anchor ACE2 to the plasma membrane could facilitate endocytosis and virus entry. In agreement with this idea, it was recently reported that ACE2 interacts directly with the cytoplasmic PDZ proteins NHERF1 and SNX27, and these interactions modulate ACE2-mediated SARS-CoV-2 cell entry [82,83]. Interestingly, both NHERF1 and SNX27 also modulate TJ organization and barrier function, by associating with the TJ protein ZO-2 (SNX27) and regulating cytoskeletal organization and membrane trafficking [84,85]. Finally, proximity label mass spectrometry analysis identifies occludin as proximal to ACE2 (ACE2 BioGrid data, [86]). Together, these observations further reinforce the notion of a mechanistic link between TJ membrane and scaffolding proteins, ACE2 and SARS-CoV-2 entry. Additional layers of regulation could be provided by the association of ACE2 with transmembrane partners, such as CD9 and ADAM17, which we identified as proteins that co-immunoprecipitate with ACE2 in lysates of cultured epithelial cells. Further experiments are required to validate the physiological relevance of the ACE2-CD9-ADAM17 complex within tissues, to examine whether depletion of either ADAM17 or CD9 affects complex formation and cellular infection by SARS-CoV-2, and to determine whether these proteins interact directly or indirectly and associate with specific PDZ proteins.

In summary, our results show that ACE2 is localized at epithelial Cell—Cell junctions and raise the hypothesis that junction-associated ACE2 could be targeted by coronaviruses to promote their entry across epithelial tissues, facilitated by inflammation through endocytosis of junctional protein complexes, and by disruption of junctional scaffolding complexes. Our data suggest a model whereby multiple protein–protein interactions, involving ACE2, ADAM17 and CD9, underlie the junctional anchoring of ACE2 and its internalization (Figure 4). Future studies should investigate the mechanisms that regulate the plasma membrane targeting, dynamic internalization and recycling of the complex, the effect of virus attachment and inflammation on ACE2 localization and internalization, and the role of ACE2-interacting junctional proteins in coronavirus entry.

## Data Availability

Data for this study are available upon request from the corresponding author, Sandra Citi (sandra.citi@unige.ch).

## Supplementary Materials

The following supporting information can be downloaded at: https://www.mdpi.com/article/10.3390/cells11040627/s1, Figure S1: Correlation of expression of ACE2 with CD9, ADAM17 occludin and cingulin.

## Abbreviations

ACE2	angiotensin-converting enzyme 2
ADAM17	a disintegrin and metalloproteinase domain-containing protein 17
AJ	adherens junction
A427	Cellosaurus A-427 (CVCL_1055)
A549	human alveolar epithelial cells
BSA	bovine serum albumin
CD9	cluster differentiation protein 9
DAPI	4′,6-diamidino-2-phenylindol
D-PBS	Dulbecco’s phosphate buffered saline
DTT	dithiothreitol
DMEM	Dulbecco’s Modified Eagle Medium
ECL	enhanced chemiluminescence
E-cadherin	epithelial cadherin
EDTA	ethylenediaminetetraacetic acid
FBS	fetal bovine serum
GFP	green fluorescent protein
GEO	Gene Expression Omnibus
HaCaT	cultured human keratinocytes
Hap1	near-haploid human cell line derived from the male chronic myelogenous leukemia (CML) cell line KBM-7
HRP	horseradish peroxidase
H1299	human non-small cell lung carcinoma cell line derived from lymph node
IB	immunoblot
IF	immunofluorescence
kDa	kilodaltons
KO	knock-out
mCCD	mouse cortical collecting duct cells
MDA-MB-231	M.D. Anderson Metastasis Breast cancer line 231
MDCK	Madin-Darby canine kidney cells
MCF-7	Michigan Cancer Foundation-7
NEAA	non-essential amino acids
PBS	phosphate buffered saline
PAGE	polyacrylamide gel electrophoresis
PDZdomain	Psd95-Dlg1-ZO-1 domain
PDZD11	PDZ domain-containing protein 11
RT	room temperature
SD	standard deviation
SDS	sodium dodecyl sulphate
ZA	zonula adhaerens
ZO-1	zonula occludens-1
