# Supplementary material for: The ACE2 Receptor for Coronavirus Entry Is Localized at Apical Cell—Cell Junctions of Epithelial Cells
Source: Cells. 2022 Feb 11;11(4):627. doi: 10.3390/cells11040627 (PMC8870730; doi:10.3390/cells11040627)
Supplement: Supplementary file 1 [file cells-11-00627-s001.zip › cells-1572709-supplementary.pdf]

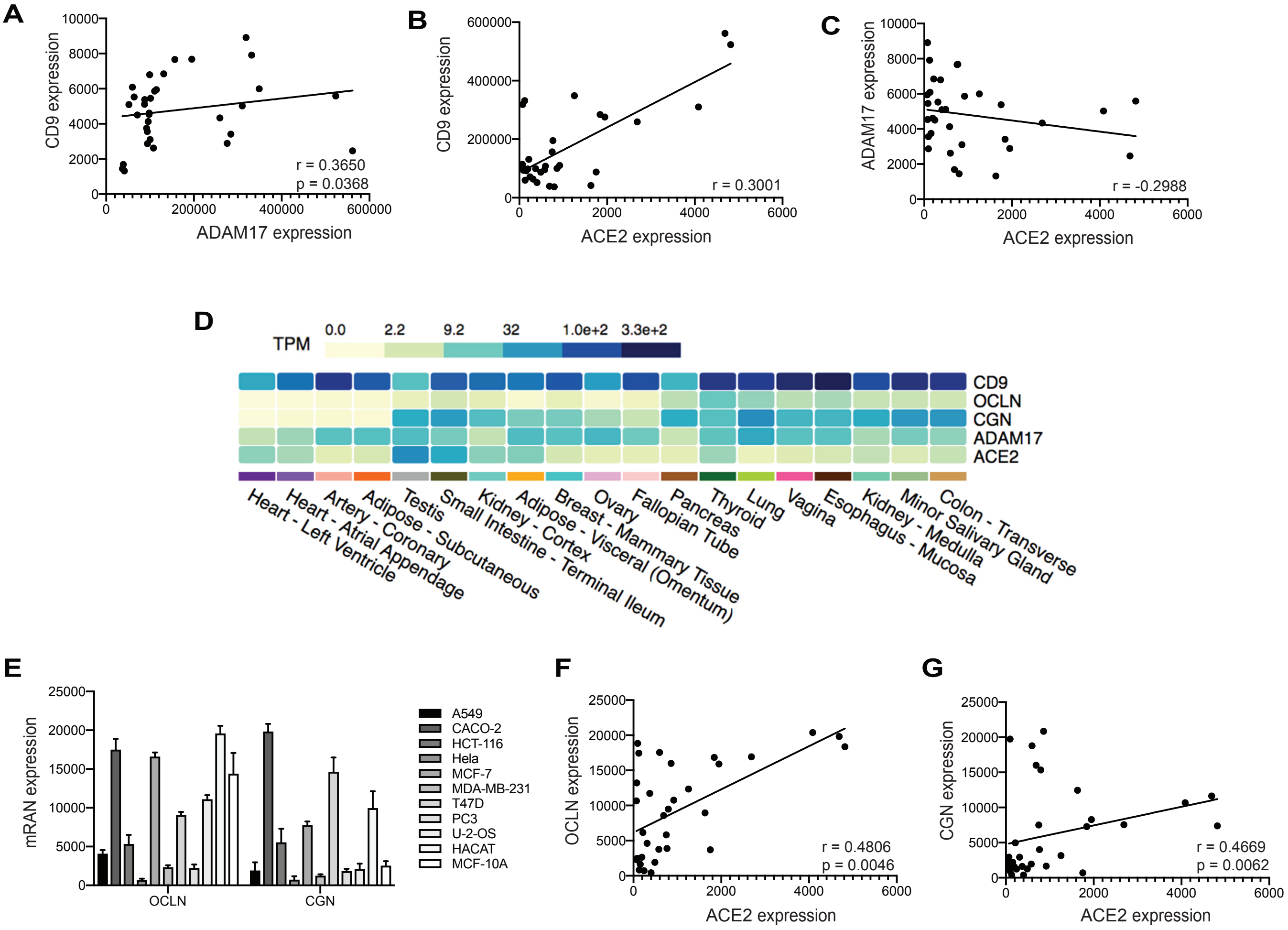

Supplementary Figure S1 (related to Figures 1-3). **Correlation of expression of ACE2 with CD9, ADAM17 occludin and cingulin**.

(A-C) Spearman's correlation analysis of mRNA expression of ADAM17/CD9 (A), ACE2/CD9 (B) and ADAM17/ACE2 (C) across the different cultured cell lines (each point represents a cell line, data from GSE41445 microarray datasets). (D) Signal intensities for mRNA expression of ACE2, CD9, ADAM17 and the TJ proteins occludin and cingulin from GTexPortal (https://ww.gtexportal.org/home/multiGeneQueryPage/ACE2,ADAM17,CD9,OCLN,CGN). (E) Signal intensities for mRNA expression of occludin and cingulin in the indicated cultured cell lines (GSE41445 microarray dataset). (F-G) Spearman's correlation analysis of ACE2 and OCLN (F) and ACE2 and cingulin (G) mRNA expression across the different cultured cell lines from GSE41445 microarray datasets.
